# The Silhouette Zoetrope: A New Blend of Motion, Mirroring, Depth, and Size Illusions

**DOI:** 10.1177/2041669517700912

**Published:** 2017-04-05

**Authors:** Christine Veras, Quang-Cuong Pham, Gerrit W. Maus

**Affiliations:** School of Art, Design and Media, Nanyang Technological University, Singapore; School of Mechanical & Aerospace Engineering, Nanyang Technological University, Singapore; Division of Psychology, School of Humanities & Social Sciences, Nanyang Technological University, Singapore

**Keywords:** zoetrope, optical illusions, anorthoscopic perception, motion, animation

## Abstract

Here, we report a novel combination of visual illusions in one stimulus device, a contemporary innovation of the traditional zoetrope, called Silhouette Zoetrope. In this new device, an animation of moving silhouettes is created by sequential cutouts placed outside a rotating empty cylinder, with slits illuminating the cutouts successively from the back. This “inside-out” zoetrope incurs the following visual effects: the resulting animated figures are perceived (a) horizontally flipped, (b) inside the cylinder, and (c) appear to be of different size than the actual cutout object. Here, we explore the unique combination of illusions in this new device. We demonstrate how the geometry of the device leads to a retinal image consistent with a mirrored and distorted image and binocular disparities consistent with the perception of an object inside the cylinder.

## Introduction

In 1834, William G. Horner first published an article about a new invention, the *Daedaleum*, a new type of optical device that would become popular much later under the name *Zoetrope*. The new apparatus was informed by previous optical illusion devices such as the Thaumatrope (1825), the Anorthoscope (invented in 1828, but only commercialized in 1836), and the Phenakistoscope (1832). Coming from a long history of inventors simultaneously working on similar ideas from different perspectives, these devices were created as both entertainment and as tools to study human vision. From Michael Faraday’s mathematical studies to Joseph Plateau’s scientific and artistic investigations, many have contributed to evolve this long lineage of optical illusions. Among them, Étienne-Jules Marey created one of the first Zoetropes to use three-dimensional figures representing the sequential phases of a bird’s flight, and Antoine Claudet pioneered devices stimulating the eyes successively to create moving stereoscopic images (Carpenter, 1868; Mannoni, 2000; Wade, 2012, 2016).

**Figure 1.**
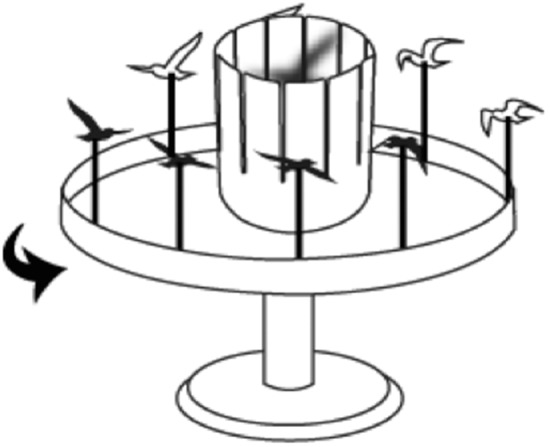
Schematic drawing of the Silhouette Zoetrope. The outside of the cylinder is painted black, whereas the inside is white (not pictured here to facilitate visualization). Cutout birds are also black and are seen as a silhouette illuminated from the back. Also see video 1, link present in the appendix.

Inspired by this history of proto-cinema optical devices in association with the Asian shadow puppet theater tradition, we present a new addition to this line of optical illusions: the “Silhouette Zoetrope.” The Silhouette Zoetrope produces the perception of animation within an empty, slotted cylinder that rotates concentrically with sequentially cutout objects placed in front of each slit (see Figure 1 and video 1, link present in the appendix). The interior of the slotted cylinder is white, while its external side and the cutout figures are black. Illuminating the inside of the cylinder enhances the effect. When the structure is rotated, an observer looking through the slits can perceive an animated silhouette moving inside the cylinder. Despite its apparent simplicity of function, the Silhouette Zoetrope compiles a remarkable set of illusions.

First, and most strikingly, the cutouts are perceived to be horizontally mirrored, that is, although the birds in Figure 1 are facing to the right, observers see the animated bird facing left when the Silhouette Zoetrope is rotated. Second, particularly with binocular viewing, the animated figure appears to be floating in the center of the cylinder. Third, the resulting animated figures may vary in size, that is, the silhouette of the bird can look smaller or larger than the physical size of the cutout depending on its placement in relation to the slit. We confirmed these observations in a set of brief experiments.

## Methods

### Participants

Eleven observers (six female, five male) volunteered to take part in this study. All had normal or corrected-to-normal visual acuity. The study was approved by Nanyang Technological University’s Institutional Review Board and was performed in compliance with the Declaration of Helsinki.

### Materials

We used an electrically driven version of the zoetrope that rotated the cylinder clockwise at a constant speed of 150 rpm. The cylinder had a radius of 4.25 cm with eight regularly placed slits, each measuring 4 mm horizontally. The figures (bird cutouts facing to the right) could be mounted in three possible positions, at 1.1 cm, 2.2 cm, or 3.3 cm distance from the cylinder in front of each slit. Observers’ viewing distance (from the center of the spinning cylinder) was about 80 cm.

### Procedure

First, the participants wore an eye patch over their nondominant eye to view the zoetrope monocularly. A movable screen covered the device and was removed only when the zoetrope was in motion. The zoetrope was setup with the cutout birds at the furthest distance, at 3.3 cm. The following questions were asked:

- “Which direction is the bird facing? Left or right?”
- “Where do you see the bird? In front of, inside, or behind the cylinder?”

Observers then removed the eye patch and answered Question (b) again for binocular viewing.

Next, we tested the perceived size of the bird in the zoetrope display. Since the animated cutouts vary in horizontal extent as the bird flaps its wings, we changed the cutouts to a static version, that is, we mounted eight identical cutouts of the bird (horizontal length 4.6 cm) outside the rotating cylinder. Observers viewed the rotating Silhouette Zoetrope with the birds mounted at one of the three distances, once each in a random order. A cardboard with five versions of the bird with different horizontal lengths (3.4, 4.0, 4.6, 5.2, and 5.8 cm) was placed in front of observers, and they were asked to indicate which of the five options most resembled their percept of the animated figure.

## Results

**Figure 2.**
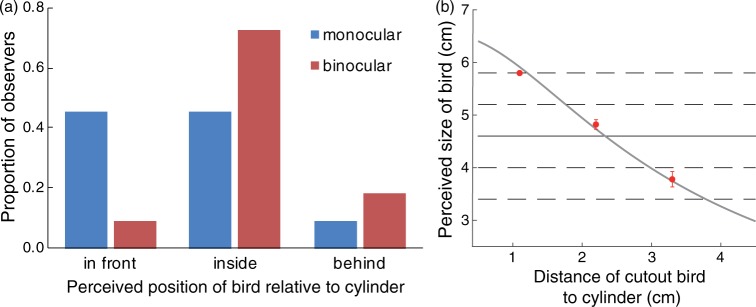
(a) Perceived position of the cutout bird relative to the cylinder. Under monocular viewing, observers were equally likely to perceive the bird in front of or inside the cylinder. Binocular viewing led to most observers seeing the bird inside the cylinder. (b) Perceived size of the bird for different mounting positions outside the cylinder (red dots, error bars denote SEM). The actual size of the bird was 4.6 cm (horizontal solid black line); observers judged size by choosing one of five size options on a cardboard in front of them (horizontal lines). The solid gray function shows predictions from a geometric model of the inside-out zoetrope (see text). Figure 2(b) shows the mean responses of 11 observers in the size estimation task (and standard errors of the mean). Perceived size varied with the cutout’s distance from the cylinder, with larger size estimates resulting when the bird was mounted closer to the cylinder. At the closest distance of cutouts to the cylinder, observers always picked the largest option (5.8 cm); at the middle distance, observers picked on average a larger option than veridical (mean = 4.82 cm, SEM = 0.09 cm); and at the furthest distance, observers picked a smaller than veridical option (mean = 3.78 cm, SEM = 0.15).

All observers (100%) reported that the bird was facing to the left, that is, in the opposite direction of the actual cutout figures placed outside the cylinder. Under monocular viewing, 45.5% of observers reported seeing the bird in front of the cylinder, whereas 45.5% saw the bird inside the cylinder. Under binocular viewing, the proportion of observers experiencing the illusion of seeing the bird inside the cylinder increased to 72.7% (Figure 2(a)).

## Discussion

In the following, we discuss the unique combination of illusions occurring in the Silhouette Zoetrope. We developed simple geometrical models to explain the perceived horizontal reversal, the perceived depth of the figure, and the distortion of perceived size.

**Figure 3.**
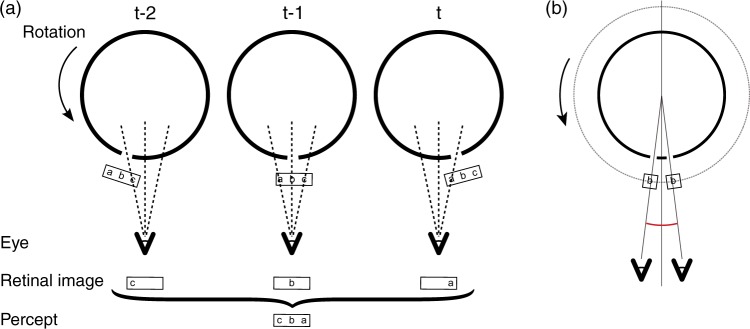
(a) The horizontal reversal of the figure can be understood by looking at three separate time points, at which the front (c), middle (b), and back (a) parts of the figure are lined up with the slit, respectively. When these three views are integrated into one percept, the figure is perceived as horizontally reversed. (b) Under binocular viewing, the resulting binocular disparities of corresponding points of the figure as the zoetrope rotates are consistent with the figure being located in the center. Just the center point of the figure is shown here for simplicity.

The apparent horizontal reversal is so counter-intuitive, that naïve observers anecdotally tried to explain the percept by hypothesizing that they were seeing the bird from *behind* the cylinder through aligned slits in the front and back. However, this was not the case. The percept of the animated figure does not change, when the slits are covered and made opaque by white paper inside the cylinder, making it impossible to view the figures on the opposite side of the cylinder through aligned slits. The reversal of the figure can be understood when considering the consecutive images presented to the retina as the cylinder rotates. With counterclockwise rotation, the rightmost part of the figure will be illuminated from the back by the slit in the cylinder first, but in the most leftmost position. Then the center of the figure is visible, and then the leftmost part of the figure in the rightmost position (see Figure 3(a)). Temporally summing over these three time points results in a horizontal reversal of the figure.

About half of observers readily perceived the image inside the cylinder, even under monocular viewing. Since there are not many other depth cues available in the display, the visual system might assume that partial visibility of the figure at any one time is caused by occlusion of the figure by the cylinder, rather than illumination of the figure from the back. Thus, the partially occluded object (the bird) would be perceived to be further away than the front of the cylinder. However, our experiment showed that binocular viewing led to a stronger illusion of the figure being perceived inside the cylinder. The perception of the bird inside the cylinder thus seems to be related to binocular disparities. In fact, matches of corresponding parts of the figure visible in both eyes are consistent with the image originating in the center of the cylinder (see Figure 3(b)).

Interestingly, the corresponding points of the figure are not available on the retina simultaneously, but sequentially, as each eye’s view of the figure is anorthoscopically “painted” onto the retina (Rock, 1981), with the images being successively perceptually constructed when the figure passes in front of the slit. Simultaneous retinal images are very dissimilar from each other; only after monocular images are temporally integrated and perceived as a whole figure can corresponding points be identified and matched stereoscopically. This is consistent with studies on the temporal properties of processing of binocular disparities (e.g., Gheorghiu & Erkelens, 2005; Kane, Guan, & Banks, 2014; Nienborg, Bridge, Parker, & Cumming, 2005; Richards, 1951).

**Figure 4.**
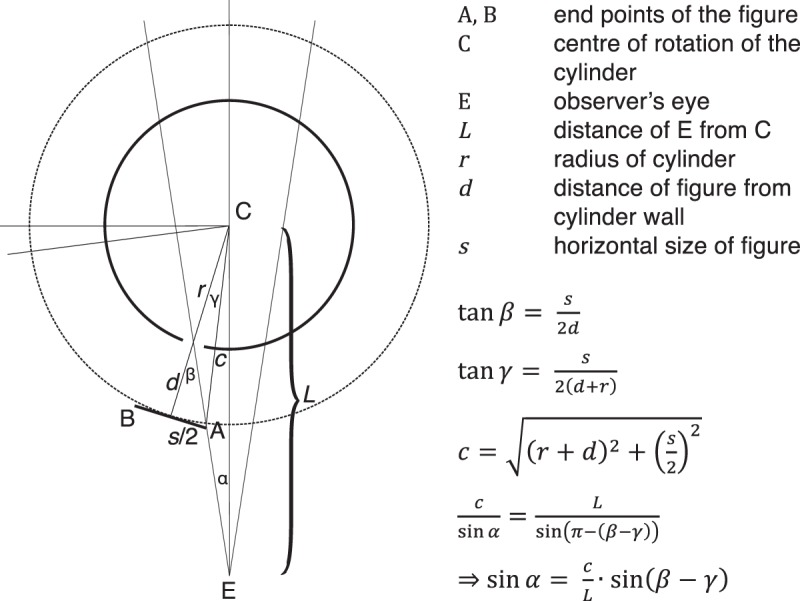
Illustration of the relevant angles and distances for working out the retinal size of the figure when the zoetrope is rotating. *β* is the angle subtended by half the figure’s length as seen from the slit, *γ* is the angle subtended by half the figure’s length as seen from C, and *c* is the distance of Point A from C and is given through the Pythagorean theorem. With *β*, *γ*, and *c*, the visual angle subtended by the figure can be calculated (see text).

The perceptual expansion or compression of the figure along its horizontal axis depends on the silhouette’s distance from the cylinder and can be explained by an analysis of the visual angle subtended by the figure.^1^ Figure 4 shows the relevant angles and distances between the viewer’s eye and the components of the zoetrope. The triangle ACE can be used to calculate *α*, the angular distance of Point A, when it is lined up with the eye and the slit, from the center of rotation C. Due to symmetry, *α* is also the angular distance of Point B when the rotation has lined it up with the slit. Thus, *α* also represents half the visual angle subtended by the figure as seen from the observer when the zoetrope is rotating. Employing the law of sines, *α* can be determined as a function of the sizes and distances in the zoetrope setup (see equations in Figure 4). Assuming that—due to the binocular constraints mentioned earlier—the observer perceives the figure in the center of the cylinder, Emmert’s law can be used to calculate the perceived size *S_perceived_* as a function of *α* and *L*:

$$S_{\mathrm{perceived}}=\mathrm{kL}2\tan\alpha$$

where *k* is a scale factor to account for any over- or underestimation of distance, as is commonly reported in distance judgments (Holway & Boring, 1941).

Using the above equation, we fitted predictions for perceived size to the empirical size judgments from our experiment (gray line in Figure 2(b)). The model predicts that perceived size of the figure decreases with increased distance between the silhouette and the cylinder, and fits the empirical data from our experiment well. Note that our model has only one free parameter, *k*. The fit in Figure 2(b) uses a value of *k = *0.77. A value of *k* < 1 indicates an underestimation of distance. Although observers overestimated distance of the figure to be inside the cylinder rather than in front, they also underestimated the total distance. This underestimation, however, might be due to a perceptual compromise between the horizontal and vertical extent of the figure when judging size.^1^

## Conclusion

We present a simple but counterintuitive innovation on the traditional zoetrope. Mounting the animated figures *outside* of the slotted cylinder still results in perception of an animated figure. On top of the illusion of motion, observers experience a unique combination of visual effects—a horizontal reversal of the figure, a mislocalization of the figure into the empty cylinder, and a misperception of the figure’s size. This simple device presents a wealth of optical and visual effects for the curious observer.

## Note

1 We only consider perceived horizontal extent of the figure here. Vertically, the figure’s angular size will be larger as the distance from the cylinder increases, that is, the distance to the observer decreases. This also means that observers perceive a distortion of the figure’s aspect ratio. However, vertical size estimates are predicted to be off by only up to 0.2 cm, whereas the horizontal size is off by up to 1.5 cm in our display (see Figure 2(b)).

## Appendix

A video of the Silhouette Zoetrope in action can be found at https://www.youtube.com/watch?v=2-A_Pcrz6xU. Please note that, obviously, the stereoscopic depth effect is not present in the video. The video corresponds roughly to the monocular condition in our experiment. Also, in the video, special lighting was used to enhance the illusion effect for the camera mainly for aesthetic reasons. However, the illusion can be perceived in practically any light conditions, as long as the contrast between the white inside of the cylinder and its darker exterior is kept.
